# Is hemolysis a novel therapeutic target in COVID-19?

**DOI:** 10.3389/fimmu.2022.956671

**Published:** 2022-08-19

**Authors:** Daiki Ousaka, Masahiro Nishibori

**Affiliations:** ^1^ Department of Pharmacology, Okayama University Graduate School of Medicine, Dentistry, and Pharmaceutical Sciences, Okayama, Japan; ^2^ Translational Research and Drug Development, Okayama University Graduate School of Medicine, Dentistry, and Pharmaceutical Sciences, Okayama, Japan

**Keywords:** hemolysis, COVID-19, sepsis, haptoglobin, hemopexin, CD163, dexamethasone, free hemoglobin

## Introduction

As of February 2022, the COVID-19 pandemic, which began at the end of 2019, has now killed 5.6 million people and infected hundreds of millions of others. One of the biggest concerns since the early days of the pandemic has been the development of drugs to control the severity of the disease, and several drugs have been proposed and are already in clinical use. However, in light of the current pandemic situation, as well as possible future pandemics, it is still important to develop novel drugs with a more diverse range of therapeutic targets. In this opinion, we focus our attention on hemolysis as a candidate target. We consider possible mechanisms underlying the relation between COVID-19 severity and hemolysis, and the feasibility of using drug candidates (or repositioning) targeting hemolysis as a protective strategy against severe COVID-19.

## Hemolysis in sepsis, including severe COVID-19-related sepsis

Many efforts have been made to treat hemolytic diseases, including congenital (e.g., sickle cell disease: SCD) and acquired (e.g., autoimmune disease, heterozygous transfusion) hemolytic diseases, which are characterized by increased destruction of red blood cells (RBC). The adverse effects of hemolysis include not only a decrease in oxygen-carrying capacity of the blood due to RBC depletion, but also the damaging activities of free hemoglobin, heme, and Fe^2+^ as damage associated molecular patterns (DAMPs). These three hemolytic products produce signals and amplify inflammation *via* toll-like receptors (TLRs) and oxidative stress. Hemoglobin causes vasoconstriction *via* nitric oxide inactivation (1). Heme has been reported to be highly toxic to many types of cells through its promotion of free radical formation and oxidative stress. On the other hand, Fe^2+^ produces hydroxyl radicals through the Fenton reaction (2), which is also harmful to the cells. In addition to the damage caused by these products, the nephrotoxicity caused by severe hemolysis can be lethal (3, 4). Recently, hemolysis has also been recognized as an aggravating factor in sepsis (5), but there is still controversy in regard to suitable targets for the treatment of hemolysis.

In the context of COVID-19, several reports have considered the potential of hemolysis as both a biomarker and therapeutic target, but to our best knowledge, no clinical trials targeting hemolysis are undergoing. Reports regarding the possibility of using hemolysis in COVID-19 as a biomarker have considered various indices of hemolysis for this purpose, including decreased hemoglobin (6, 7), elevated free heme (8), increased RBC distribution width (RDW) (9), high ferritin, high lactate dehydrogenase (10, 11), and high bilirubin (12). Heme oxygenase-1 (HO-1) induction (13), and supplementation of haptoglobin and hemopexin (14) have anti-inflammatory effects *via* scavenging and clearance of hemolysis-related degradation products. In addition, it has been reported that protecting RBCs against hemolysis is important for the proper functioning of the central nervous system (15). Moreover, the increasing risk is reported in the case of COVID-19 infection in patient with glucose-6-phosphtase dehydrogenase (G6PD) deficiency, who usually have structurally vulnerable RBCs which are easy to hemolysis, has been suggested (16). There are also reports on changes in structural proteins and membrane lipids remodeling in RBCs in COVID-19 patients (17). From these data, both targeting hemolysis-related products and protection of RBCs are considered to be reasonable therapeutic strategies to protect against the development of severe COVID-19.

Many of the mechanisms in terms of COVID-19 related hemolysis are thought to be similar to those reported in the pathogenesis of sepsis (e.g., mechanisms involving inflammation, complement activation, autoantibodies) (18). However, mechanisms specific for COVID-19 have also been proposed, for example, viral invasion of the SARS-CoV-2 virus into RBCs (through ACE2, CD147, CD26), which then undergo hemolysis (19–21). Regarding viral infection to RBCs, binding of virus and heme may lead to resistance against the anti-viral effect of heme. In other words, a component protein of SARS-CoV2, such as open-reading frame 8, may bind to heme, resulting in decreased anti-viral activity of heme-induced miRNA processing (22). In fact, a study using a computational-experimental approach reported a direct binding between SARS-CoV2 and heme (23). Interestingly, there are many viruses that have been reported to have an affinity for RBCs (e.g., hepatitis C and B, Ebola, human immunodeficiency virus (HIV), dengue, and Zika) (24–28). On the other hand, there is a report of 6 patients with paroxysmal nocturnal hematuria (PNH) who developed severe hemolysis after the vaccine for COVID-19 (29), which implies that some autoantibodies against SARS-CoV2 component proteins may cause hemolysis (30).

In addition to hemolysis, a recent study has conjectured that increased erythrophagocytosis may cause the decrease in hemoglobin in COVID-19 patients (31). In this case, the mechanism would involve TLR9 on the surface of RBCs binding to mitochondrial CpG, which would lead to decreased RBC deformability and erythrophagocytosis by macrophages (31). This suggests that RBCs are “immune sentinels” that work at the forefront of the immune system (32). In other words, RBCs block the invasion of viruses, including SARS-CoV2, and thus any entry of the virus into RBCs would be part of a biological defense response rather than a passive invasion.

Lastly, we should consider the relationship between COVID-19 and previously reported severity factors, including hypertension, obesity, diabetes, and thromboembolism caused by neutrophil extracellular traps (NETs) (33–35). Interestingly, hemolytic products such as free heme have been shown to induce NETs as DAMPs (36, 37). Thus, hemolysis could be a marker of disease progression, and a therapeutic target.

## Therapeutic mechanisms of dexamethasone in COVID-19

One of the reasons why we focus on the importance of hemolysis in COVID-19 is that dexamethasone is known to have a beneficial effect in the treatment of COVID-19 (38–41). Dexamethasone is one of the earliest therapeutic agents to be applied clinically, as its efficacy was demonstrated in several clinical trials in the early stages of COVID-19. However, its therapeutic mechanism is still not fully understood (42). Surprisingly, although corticosteroids, including dexamethasone, have long been used clinically for sepsis (mainly bacterial sepsis), the results of multiple trials have reported that efficacy of corticosteroids cannot be proven until now (43, 44). Whether viral or bacterial (or fungal), the pathways of the sepsis cascade containing dysregulated innate immune responses are similar in many respects, but the effect of dexamethasone was clearly demonstrated in COVID-19. We hypothesize that an acceleration in the scavenging of hemolytic products by dexamethasone may be one of the therapeutic mechanisms. In fact, corticosteroid therapy has been used for decades in patients with SCD and PNH, which are typical hemolytic diseases (45–47). Corticosteroids have a wide range of pharmacological effects, but the main one is a strong immunosuppressive effect *via* the glucocorticoid receptor, and one corticosteroid, dexamethasone, is known to promote scavenging of hemolytic products by inducing CD163 receptor expression (48–51). The CD163 receptor is widely known as a scavenger receptor for the hemoglobin-haptoglobin complex, which is abundantly expressed in macrophages (52). In the pathogenesis of COVID-19, the CD163 induction effect of dexamethasone may suppress the deteriorated inflammation by hemolytic products such as free hemoglobin. Since sepsis is a disease condition involving a wide spectrum of pathological processes, the degree of involvement of hemolysis may vary greatly in each individual. In the case of COVID-19-induced sepsis, it is possible that hemolysis might be an exacerbating factor for transition from mild to severe COVID-19 based on the clinical examination data.

Another study reported that dexamethasone was effective in a group of COVID-19 patients who were ventilated or oxygenated, while there was no reduction in mortality in the patients without oxygen (41). A possible explanation for this may be hemolytic toxicity *via* free radical generation by high oxygen concentration, or ventilator-related hemolysis. On the other hand, in the group of patients whose blood oxygen saturation was so low that they required a ventilator or oxygen, hemolysis at the alveolar level (leakage of RBC into the alveolar space) was prominent, suggesting that the hemolysis-scavenging effect of dexamethasone was of great benefit. In any case, it is clear that dexamethasone is effective for preventing mortality in patients with COVID-19. By setting a new target of scavenging hemolytic products, it would be possible to consider a combination therapy with anti-hemolytic serum proteins, such as haptoglobin and hemopexin, to maximize the efficacy of CD163 upregulation by dexamethasone.

## Impaired scavenging hemolytic products under inflammatory conditions

The mechanism of hemolysis in the pathogenesis of severe infections, the amplification of inflammation by hemolytic products and the direct cytotoxicity of those products have already been extensively investigated (2, 18). In this section, we discuss the impaired efficacy of scavenging hemolytic products under the inflammatory condition, which may be a new therapeutic target.

There are two main processing systems for hemolysis: a free hemoglobin processing system using haptoglobin (52), and a free heme processing system by hemopexin (53). Both systems function by endocytosis *via* scavenging receptors (CD163 and CD91, respectively) of macrophages and detoxification of hemolytic products by a degradation and recycling system thorough HO-1. It is clear that these physiological processing systems for hemolysis are depleted or fail to adapt to acute massive hemolysis or chronic persistent hemolysis. Therefore, we focus on the hemolysis in COVID-19 and consider the change in scavenging of hemolysis-related products under severe inflammation and tissue destruction environment. In particular, in the environment of ARDS, which is a major cause of death in COVID-19, increased vascular permeability due to inflammation is evident. ARDS is also associated with the impairment of vascular endothelial cells, which causes RBC leakage, hemolysis, and amplification of inflammation in the extravascular spaces. Furthermore, increased dead cells as well as overloaded macrophages by hemoglobin could cause a change in the microenvironment, including the pH, and could increase the release of some metal ions from platelets and activated mast cells (54, 55). These factors may be responsible for the delayed scavenging of hemolytic products. Thus, the therapeutic targets for hemolysis in sepsis may include preservation of the CD163 scavenger system, such as chelation for excessive metal ions in the microenvironment in addition to the usual therapeutic strategies. Indeed, shedding of the CD163 receptor occurs under sepsis-mimicking conditions (e.g., TLR4 stimulation by lipopolysaccharide) (56). Furthermore, during treatment of acute myelogenous leukemia with gemutuzumab ozogamicin (a CD33-targeted antibody-conjugate), CD163-positive macrophages are impaired and the scavenger ability for hemolysis-related products is significantly reduced (57, 58). In summary, it is necessary to consider the possibility that the impaired scavenging function of CD163-positive macrophages may occur under specific conditions.

## Discussion

**Figure 1:**
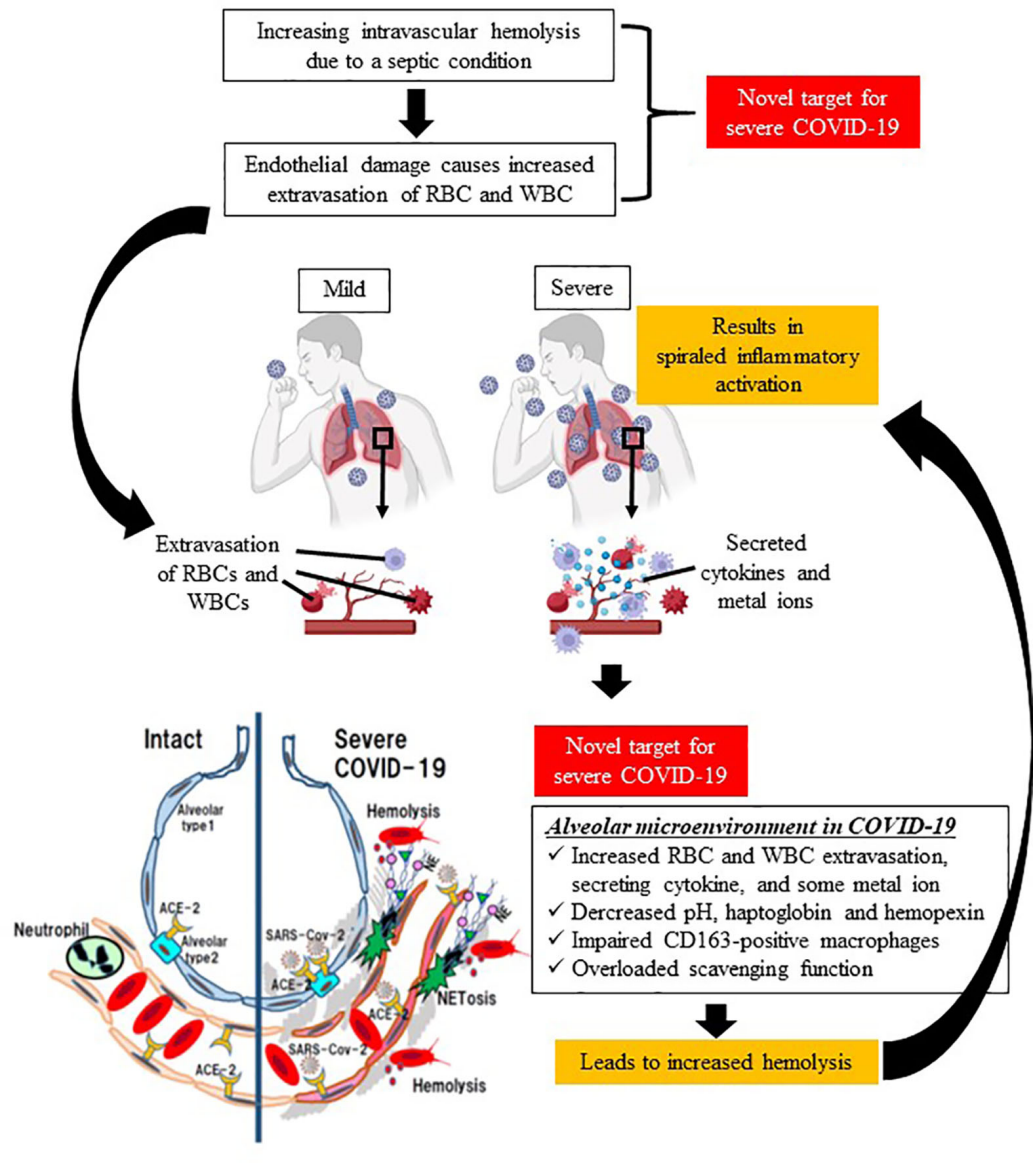
Schematic diagram of spiraled inflammatory activation and tissue damage by hemolysis in severe COVID-19. Sepsis causes intravascular hemolysis by many mechanisms (e.g., microvasculitis, complement activation, glucose metabolism, and eryptosis), leading to increased vascular permeability, and thereby extravasation of both red blood cells and immune system cells. The first target in the proposed treatment strategy is a decrease in intravascular hemolysis. In terms of the alveolar microenvironment, increases in extravasated cells, cytokines, and metal ions deteriorate hemolysis in the extracellular space, which is associated with spiraled inflammatory activation in lung issue. Therefore, the second therapeutic target is a decrease in extravascular hemolysis. (This figure was created at BioRender.com.).

Typical hemolytic diseases include SCD and thalassemia caused by hereditary β-globin abnormality, and acquired hemolytic anemias such as autoimmune hemolytic disease, PNH, and mechanical hemolysis caused by cardiopulmonary bypass. In recent years, although it has been recognized that hemolysis is also a factor in the severity of sepsis, no anti-hemolysis therapy has been used in clinical settings. In this opinion, we propose hemolysis as a new therapeutic target in COVID-19, because severe COVID-19 is a viral sepsis with a characteristic course of rapid deterioration of ARDS, and thromboembolism with suspected NETs (**Figure 1**).

The mechanism of hemolysis in COVID-19 is thought to be similar to that in sepsis (microangiitis, vascular occlusion, eryptosis, membrane deformability, complement activation, hypoglycemia, etc.) (5), but some unique factors have been identified. These factors include direct RBC destruction by SARS-CoV2 (17, 20, 21) and autoantibody-induced hemolysis against viral proteins (30). The accumulation of hemolytic products due to increased hemolysis and delayed scavenging of hemolytic products may amplify the inflammatory response as DAMPs or aggravate the condition through direct tissue damage, which may be the mechanism leading to the severity of COVID-19.

Finally, in this opinion, we addressed the relevance of hemolysis as a novel therapeutic target to reduce the severity of COVID-19. It is necessary to verify hemolysis as a new target and accumulate knowledge in order to alleviate the current situation and prepare for future pandemics.

## Author contributions

All authors listed have made a substantial, direct, and intellectual contribution to the work and approved it for publication.

## Funding

This research was supported by AMED under Grant Number JP19im0210109h0003.

## Conflict of interest

Author MN been an advisor of the Japan Blood Products Organization and obtains the research support from the company.

The remaining author declares that the research was conducted in the absence of any commercial or financial relationships that could be construed as a potential conflict of interest.

## Publisher’s note

All claims expressed in this article are solely those of the authors and do not necessarily represent those of their affiliated organizations, or those of the publisher, the editors and the reviewers. Any product that may be evaluated in this article, or claim that may be made by its manufacturer, is not guaranteed or endorsed by the publisher.
